# Inhibition of *Leishmania (Leishmania) amazonensis* and Rat Arginases by Green Tea EGCG, (+)-Catechin and (−)-Epicatechin: A Comparative Structural Analysis of Enzyme-Inhibitor Interactions

**DOI:** 10.1371/journal.pone.0078387

**Published:** 2013-11-08

**Authors:** Matheus Balduíno Goncalves dos Reis, Letícia Correa Manjolin, Claudia do Carmo Maquiaveli, Osvaldo Andrade Santos-Filho, Edson Roberto da Silva

**Affiliations:** 1 Programa de Iniciação Científica da Faculdade de Zootecnia e Engenharia de Alimentos, Universidade de São Paulo, Pirassununga, São Paulo, Brazil; 2 Programa de pós-graduação em Fisiologia, Departamento de Fisiologia, Universidade de São Paulo, Faculdade de Medicina de Ribeirão Preto, Ribeirão Preto, São Paulo, Brazil; 3 Laboratório de Modelagem Molecular, Departamento de Síntese Orgânica, Farmanguinhos/Fundação Oswaldo Cruz, Rio de Janeiro, Rio de Janeiro, Brazil; 4 Departamento de Medicina Veterinária, Faculdade de Zootecnia e Engenharia de Alimentos, Universidade de São Paulo, Pirassununga, São Paulo, Brazil; Casey Eye Institute, United States of America

## Abstract

Epigallocatechin-3-gallate (EGCG), a dietary polyphenol (flavanol) from green tea, possesses leishmanicidal and antitrypanosomal activity. Mitochondrial damage was observed in *Leishmania* treated with EGCG, and it contributed to the lethal effect. However, the molecular target has not been defined. In this study, EGCG, (+)-catechin and (−)-epicatechin were tested against recombinant arginase from *Leishmania amazonensis* (ARG-L) and rat liver arginase (ARG-1). The compounds inhibit ARG-L and ARG-1 but are more active against the parasite enzyme. Enzyme kinetics reveal that EGCG is a mixed inhibitor of the ARG-L while (+)-catechin and (−)-epicatechin are competitive inhibitors. The most potent arginase inhibitor is (+)-catechin (IC_50_ = 0.8 µM) followed by (−)-epicatechin (IC_50_ = 1.8 µM), gallic acid (IC_50_ = 2.2 µM) and EGCG (IC_50_ = 3.8 µM). Docking analyses showed different modes of interaction of the compounds with the active sites of ARG-L and ARG-1. Due to the low IC_50_ values obtained for ARG-L, flavanols can be used as a supplement for leishmaniasis treatment.

## Introduction

Food polyphenols show bioactivities that contribute to human health [1], [2]. Balanced food intake enriched with polyphenols from vegetables, green tea, wine and fruits can prevent cardiovascular diseases [3]. In addition to the known antioxidant activity attributed to green tea (−)-epigallocatechin-3-gallate (EGCG), this compound paradoxically contributes to lethal mitochondrial damage in *L. (L.) amazonensis* [4]. EGCG is also active against *Leishmania (Leishmania) donovani*, *Trypanossoma brucei rhodesiense* [5] and *Trypanosoma cruzi* [6].

Enzymes of the polyamine (PA) synthesis pathway are considered to be important targets for drug development against leishmaniasis [7],[8]. PAs have a central role in proliferation, differentiation, and antioxidant mechanisms in *Leishmania* [8], [9]. Antioxidant mechanisms in trypanosomatids use the PA spermidine to synthesize trypanothione. Trypanothione protects the parasite from oxidative stress by promoting the removal of reactive nitrogen species [10], reactive oxygen species [11] and other reactive species produced by the host's defense system.

Arginase is the first enzyme of the PA pathway and was considered a target to control *Leishmania* infection. Arginase from *Leishmania* (ARG-L) is localized in glycosomes and may be essential for the physiological rhythm of the parasite; it is involved in a complex balance that defines the fate of L-arginine [12]. The roles of arginases in infection were studied in mutants containing a knockout of ARG-L gene [13], a mutation resulting in ARG-L localized in the cytosol instead of in the glycosome organelles [12], and in an arginase null host [14].

Mammals have two arginases: ARG-1 and ARG-2 that are localized in the cytosol and mitochondria, respectively. An increased level of arginase is correlated with a decreased level of NO because arginase and nitric oxide synthase use the same substrate, L-arginine. Human arginase is increased in HIV patients co-infected with leishmaniasis [15] and in lesions of cutaneous leishmaniasis [16]. Due to the increased arginase activity in patients with visceral leishmaniasis, arginase was proposed as a marker of infection [17].

ARG-L and ARG-1 are used as targets for controlling *Leishmania* infection by blocking both parasite and host arginase [18], [19]. In this study, we have tested the flavanols EGCG, (+)-catechin and (−)-epicatechin against arginase from *Leishmania amazonensis* (ARG-L) and against rat liver arginase (ARG-1). In addition, the docking simulation of the interaction between inhibitors and the structural model of ARG-L allowed a visualization of the profile of interaction of dietary flavanols with the catalytic site of the enzyme.

## Materials and Methods

### Materials

(+)-catechin, (−)-epicatechin, EGCG, gallic acid, MnSO_4_, L-arginine, CelLytic B, MOPS (4-morpholinepropanesulfonic acid), CHES (2-(cyclohexylamino)ethanesulfonic acid), PMSF (phenyl-methyl-sulfonyl fluoride), yeast extract and tryptone were purchased from Sigma-Aldrich. Reagents for urea analysis were purchased from Quibasa (Belo Horizonte, MG, Brazil).

### Expression and purification of arginases

Recombinant ARG-L was expressed as native protein as described previously [20]. To obtain liver to prepare rat liver arginase (ARG-1), one animal was anesthetized with sodium thiopental (40 mg/kg, i.p.) and after liver procured the animal was killed via anesthesia overdose. Rat liver arginase (ARG-1) was prepared by lysing 5 g of liver cells in a 100 mL buffer containing 100 mM Tris and 1 mM EDTA using a blender. The homogenate was centrifuged at 5000×*g*, and pigments in the supernatant were removed by liquid chromatography (open column) using 5 mL of Sepharose Chelating resin (GE Healthcare) charged with Ni^+2^. The resulting arginase solution was fully activated by heat at 60°C in the presence of 10 mM of MnCl_2_ [21]. Following activation, the solution was centrifuged at 20000×*g*, and the clarified extract was used to test arginase inhibition. The experiments were performed in accordance with the ethical principles for animal experimentation adopted by the Brazilian College of Animal Experimentation, and the Animal Experimentation Committee of the University of São Paulo at Ribeirão Preto School of Medicine approved the study protocol (COBEA/CETEA/FMRP-USP, protocol no.050/2010).

### Inhibitor Screening and determination of IC_50_

Inhibition analyses of ARG-1 and ARG-L were performed in 50 mM CHES buffer (pH 9.5) in the presence L-arginine (pH 9.5) and variable or fixed concentrations of inhibitors. The reaction mixtures were incubated at 37°C for 15 minutes, and urea production was determined by the Berthelot method [22].

Concentrations that inhibited 50% of the catalytic activity of the enzyme (IC_50_) were obtained by serial dilution of inhibitor (1∶10) with 50 mM of L-arginine in CHES buffer, 50 mM, pH 9,5. The mathematical sigmoidal model (log IC_50_) was used to calculate the IC_50_ using Origin 8.0 software. All experiments were performed in duplicate in at least three independent experiments. For the IC_50_, the coefficients of nonlinear regression used in the calculation were R^2^≥0.95.

### Determination of the constants Ki, Ki' and the mechanism of inhibition

All reactions were performed in CHES buffer, 50 mM, pH 9.5, containing various concentrations of the substrate L-arginine (12.5, 25, 50 and 100 mM) at pH 9.5. Inhibitors were used at three different concentrations similar to the IC_50_. The different substrate and inhibitor concentrations were obtained by serial dilution. A mixture, M1, containing L-arginine (pH 9.5) at double the desired concentration and a second mixture, M2, containing the enzyme (2000 units) diluted in 125 mM CHES buffer (pH 9.5) were prepared. The reaction mixture was obtained by homogenizing 50 µL of M1, 10 µL of inhibitor and adding 40 µL of M2. The addition of M2 was synchronized every 15 seconds, followed by immediate incubation in a water bath for 15 minutes at 37°C. The urea produced was analyzed as described above.

The constant Ki was determined for inhibitors that showed competitive and mixed inhibitors while Ki' was determined for inhibitor that showed noncompetitive inhibition and mixed inhibition [23], [24]. For inhibitors that exhibited noncompetitive inhibition, where Ki is equal to Ki', the two graphs (Dixon plot and Cornish-Bowden plot) were used in the calculation. Each constant was determined by calculating X values at the intersection points between two lines obtained by linear regression. For noncompetitive inhibition, y = 0 for any equation to find the values of the constants Ki and Ki'. Ki and Ki' is a positive value of the calculated X (Ki = −X).

The equation for the Dixon (1) and Cornish Bowden (2) plots are:
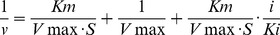
(1)

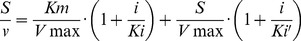
(2)


In equations (1) and (2), 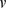 is the rate of the reaction, 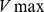
is the maximum rate of the reaction, 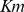 is the Michaelis constant, 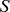 is the substrate concentration, 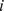 is the inhibitor concentration, 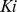 is a dissociation constant of the enzyme-inhibitor (EI) complex and 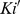 is the dissociation constant of the enzyme-inhibitor-substrate (EIS) complex.

For three different values of S, three lines were drawn for 1/v against i (Dixon plot) and three lines for S/v against i (Cornish-Bowden plot). In the Dixon plot, the lines intersect at a point where i  = −Ki and 1/v =  [1- (Ki/Ki')]/V_max_; in the Cornish-Bowden plot, they intersect at a point where i = −Ki' and S/v = K_M_[1- (Ki'/Ki)]/V_max_ [24].

To make wizard Ki and Ki' calculations, linear data fitting was performed using Microsoft Office Excel v.2013. The linear equations were obtained by linear regression R^2^>0.85. Linear regression retrieved the linear equation y_(n)_ = a_(n)_x_(n)_ + b_(n)_, which calculates the reciprocal of the ratio of reaction 1/v (y_(n)_) as a function of variable inhibitor concentration (x_(n)_). The term b(n) is the reciprocal of the maximum ratio of reaction (1/V_max_) obtained at a fixed S concentration and variable i concentration.

The general equation used to calculate Ki and Ki' from the experimental data was compiled by setting y_(1)_ = y_(2)_ as follows:
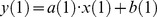
(3)


for S_(1)_, and
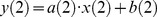
(4)


for S_(2)_

At the intersection point 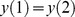 and 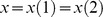 we obtained:
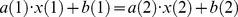
(5)


the resulting x value is:
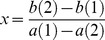
(6)


Finally, because x = i, Ki = −i (Dixon plot), Kí^1^ = −i (Cornish-Bowden plot), the equations used to calculate the dissociation constants K (Ki or Ki') were similar; both use “a” (the angular coefficient) and “b” (the linear coefficient) of two distinct straight lines from the experimental data:
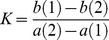
(7)


The Dixon and Cornish-Bowden plots were constructed using a mean of each point obtained from three independent experiments performed in duplicate. Three interception points were obtained by the combination of two linear equations for each time point. Then, Ki and Ki' were calculated using equation (7) and were expressed as mean values ± SEM (standard error of the mean).

### Molecular modeling

The target compounds (Table 1) were modeled *in silico*, and energy minimization was performed over 1,000 steps using the steepest descent method, Gasteiger-Hückel charges, a dielectric constant of 80, and the Tripos force field. The structures were further optimized using the conjugated gradient method.

**Table 1:** Inhibition of the *L. (L.) amazonensis* arginase by natural compounds: IC_50_, dissociation constant, docking energy and mode of enzyme inhibition.

Ligand	1IC_50_ (µM)	Ki (µM)	Docking (kcal/mol)	Mode of inhibition
(+)-catechin^a^	0.77±0.01	12.0±2.5	−99.41	Competitive
(−)-epicatechin^b^	1.8±0.5	3.0±0.4	−79.35	Competitive
EGCG^c*^	3.8±0.1	4.0±0.5	−129.27	Mixed
Gallic acid^b, d^	2.2±0.2	7.2±1.4	−66.21	Noncompetitive

1 Data are expressed as the mean ± SEM. IC_50_ differs for a compound without a common letter (p<0.05). ^*^ EGCG  =  (−)-Epigallocatechin-3-gallate.

Ligand-enzyme docking simulations were performed using the molecular docking algorithm MolDock and the Molegro Virtual Docker 4.3.0. MolDock uses a heuristic search algorithm (*i.e*., guided differential evolution), which combines differential evolution and a cavity-prediction algorithm. The docking scoring function is an extension of the piecewise linear potential (PLP).

For the target enzymes analyzed in this study, a previously constructed comparative model of ARG-L [25] and an ARG-1 crystal structure (PDB: 1RLA) were used. The docking simulations [26] were carried out by applying the MolDock algorithm [27], which was implemented in the Molegro Virtual Docker software (Molegro Virtual Docker 4.0).

### Data analysis

Statistical analysis was performed using an ANOVA, a posteriori Tukey tests and Graph Pad Prism 4.0. For all tests, values of p<0.05 were considered significant.

## Results

### Determination of Ki, Ki', the mechanism of ARG-L inhibition and IC_50_

**Figure 1:**
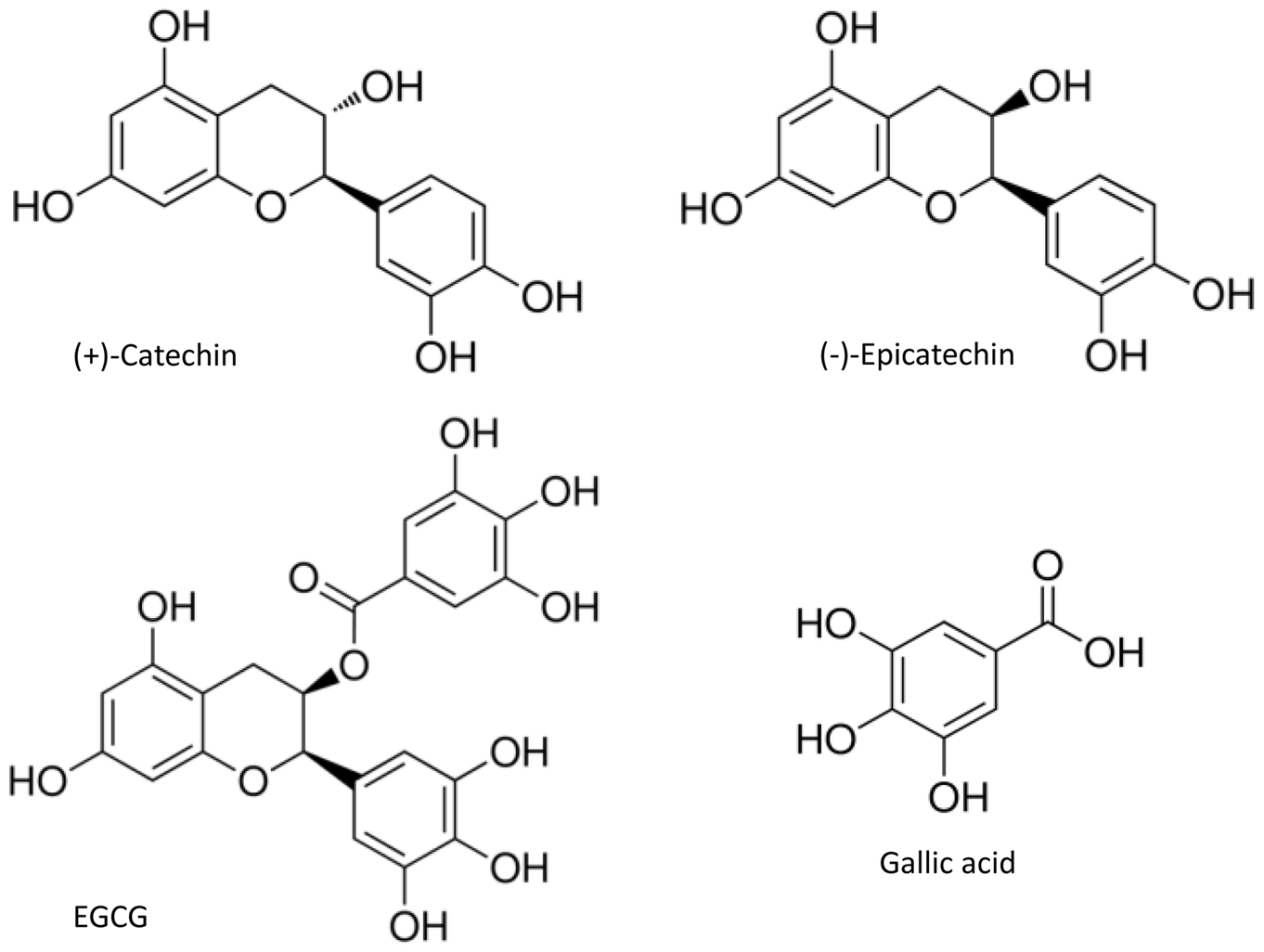
Compounds.

**Figure 2:**
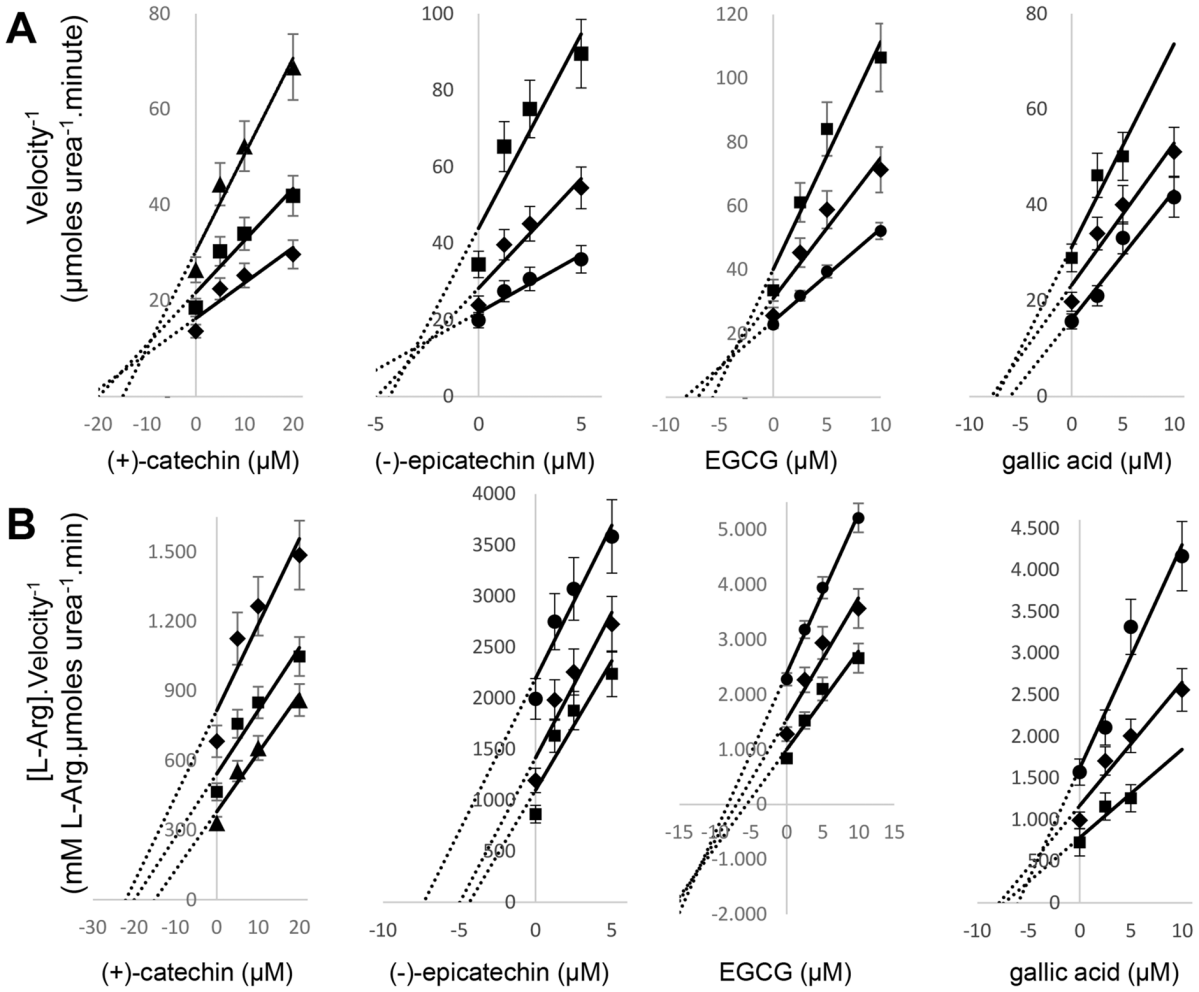
The mechanism of arginase inhibition by flavanols. The Ki constants were measured using Dixon plots (A), and the Ki' constants were determined by a Cornish-Bowden plot (B). EGCG is a mixed inhibitor (Ki≠Ki') and gallic acid is noncompetitive inhibitors (Ki = Ki'), whereas (+)-catechin and (−)-epicatechin are competitive inhibitors (Ki'>>>Ki). The concentrations of L-arginine used were 100 mM (•), 50 mM (♦), 25 mM (▪) and 12.5 mM (▴). The inhibitor concentrations were varied from 1.25 to 20 µM. Each point drawn represents the mean of three independent experiments (n = 3) performed in duplicate. Error bars show the standard error of the mean.

The mechanism of enzyme inhibition was determined for the four compounds (Figure 1) using Dixon plots [23] and Cornish-Bowden plots [24]. Ki and Ki' are the inhibition constants for the binding of the inhibitor to the enzyme and to the enzyme-substrate complex, respectively. The constants of inhibition Ki and Kí are given by the intersection points of straight lines in the Dixon and Cornish-Bowden plots, respectively. The compounds (+)-catechin (Ki = 12.0±2.5 µM) and (−)-epicatechin (Ki = 3.0±0.4 µM) have been shown to be competitive inhibitors with intersection points above the i axis in the Dixon plot and parallel lines in the Cornis-Bowden plot (Ki'→∞), indicating that Ki'>>>Ki (Figure 2). EGCG is a mixed inhibitor, because the intersection point occurred above the i axis in the Dixon plot and below the axis in the Cornish-Bowden plot (Figure 2). For EGCG, the Ki (4.0±0.5 µM) differed significantly (p<0.001) from the Ki' (12.5±0.4 µM). For gallic acid, the dissociation constants Ki and Ki' did not differ significantly (p>0.05); it is considered a noncompetitive inhibitor, with Ki = Ki' = 7.2±1.4 µM. Table 1 shows the IC_50_ values obtained by nonlinear regression and the Ki values obtained with the Dixon and Cornish-Bowden plots (Figure 2).

### Inhibition of ARG-1 by polyphenols

In order to investigate the differences between inhibition of ARG-L and inhibition of mammalian arginase we tested inhibitors against rat liver arginase (ARG1). EGCG, (+)-catechin, (−)-epicatechin and gallic acid did not significantly inhibit ARG-1 at 10 µM or 100 µM. Thus, the assay of ARG-1 activity was performed using inhibitors at 1000 µM. The strongest inhibition was observed for EGCG (29%), followed by (+)-catechin (26%), (−)-epicatechin (22%) and gallic acid (20%). At 1000 µM the compounds failed to inhibit 50% of activity of the enzyme; therefore, the IC_50_s can be considered to be greater than 1000 µM, *i.e.,* at least 250 times greater than the IC_50_ obtained for ARG-L inhibition. The maximum IC_50_ for ARG-L is estimated to be 3.8±0.1 µM (for EGCG). These results indicate that these four compounds are potent and selective inhibitors of ARG-L.

### Comparative structural analysis of arginase-inhibitor interactions

**Figure 3:**
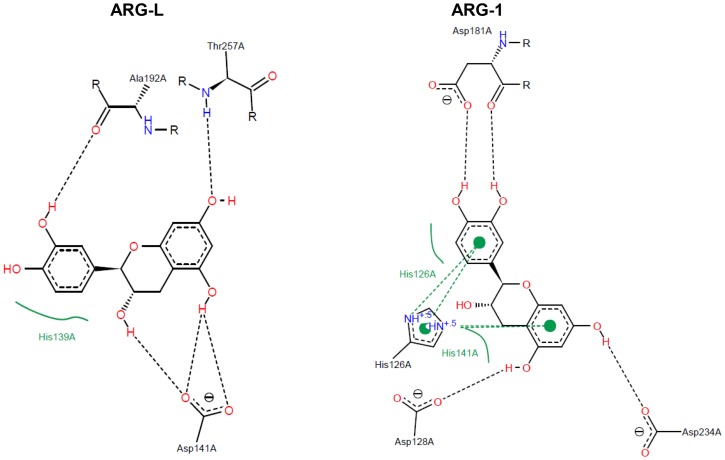
Docked (+)-catechin in the binding site of arginases. Ala192, Asp141 and His139 in ARG-L occupy the same positions in the primary structure as Asp181, Asp128 and His126 in ARG-1.

**Figure 4:**
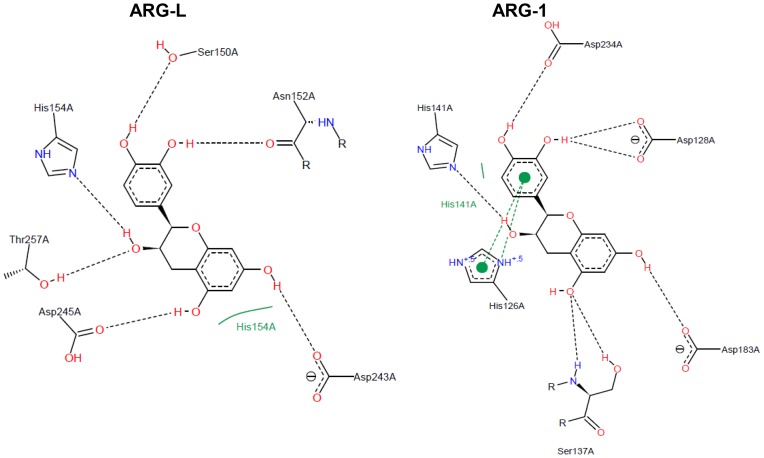
Docked (−)-epicatechin in the binding site of arginases. Ser150, His154 and Asp245 in ARG-L occupy the same positions in the primary structure as Ser137, His141 and Asp234 in ARG-1.

**Figure 5:**
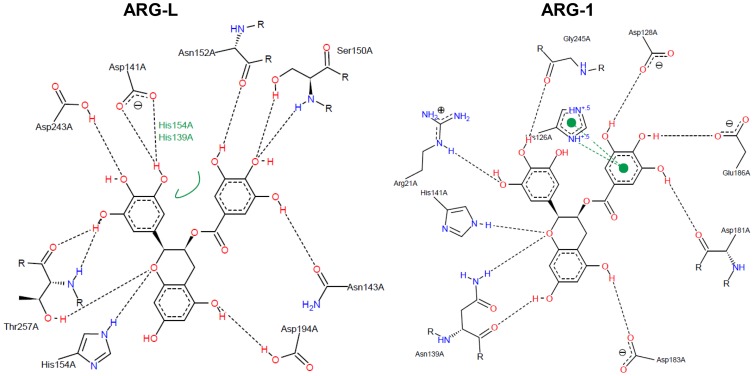
Docked (−)-epigallocatechin-3-gallate in the binding site of arginases. His139, Asp141, Asn152, His154 and Asp194 in ARG-L occupy the same positions in the primary structure as His126, Asp128, Asn139, His141 and Asp183 in ARG-1.

The docking scores of the interactions between the arginase from *L. amazonensis* and the target compounds are shown in Table 1. Figure 3, 4, 5, and 6 show a 2D-representation of the flavanoid-enzyme interactions. The intermolecular hydrogen bonds are shown as black dashed lines, and the hydrophobic interactions are shown as continuous green lines. The hydrogen bonds serve as “molecular anchors” for binding the compounds to the enzyme active site.

**Figure 6:**
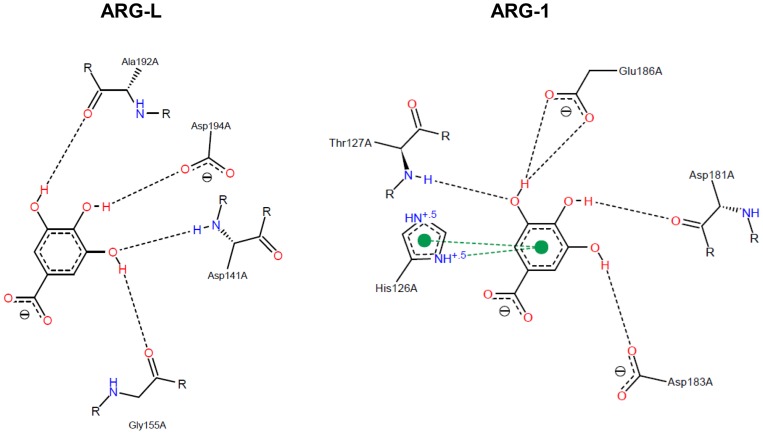
Docked gallic acid in the binding site of arginases. Ala192 and Asp194 in ARG-L occupy the same positions in the primary structure as Asp181 and Asp183 in ARG-1.

A 2D view shows that the interaction of (+)-catechin with ARG-1 occurs at a 90° clockwise position compared with ARG-L (Figure 3). The ARG-L backbone carbonyl from Ala192 and the carboxylic radical residue from Asp141 donate hydrogen bonds (H-bonds) to (+)-catechin, whereas the catechol group receives an H-bond from Thr257 (ARG-L numbering). The ARG-1 interaction with (+)-catechin is characterized by H-bond donations to Asp128, Asp 234 and Asp181 (ARG-1 numbering). Ala192 and Asp141 in ARG-L occupy the same positions in the primary structure as Asp181 and Asp128 in ARG-1. His139 (ARG-L) and its equivalent His126 (ARG-1) interact hydrophobically with the catechol group of (+)-catechin.

**Figure 7:**
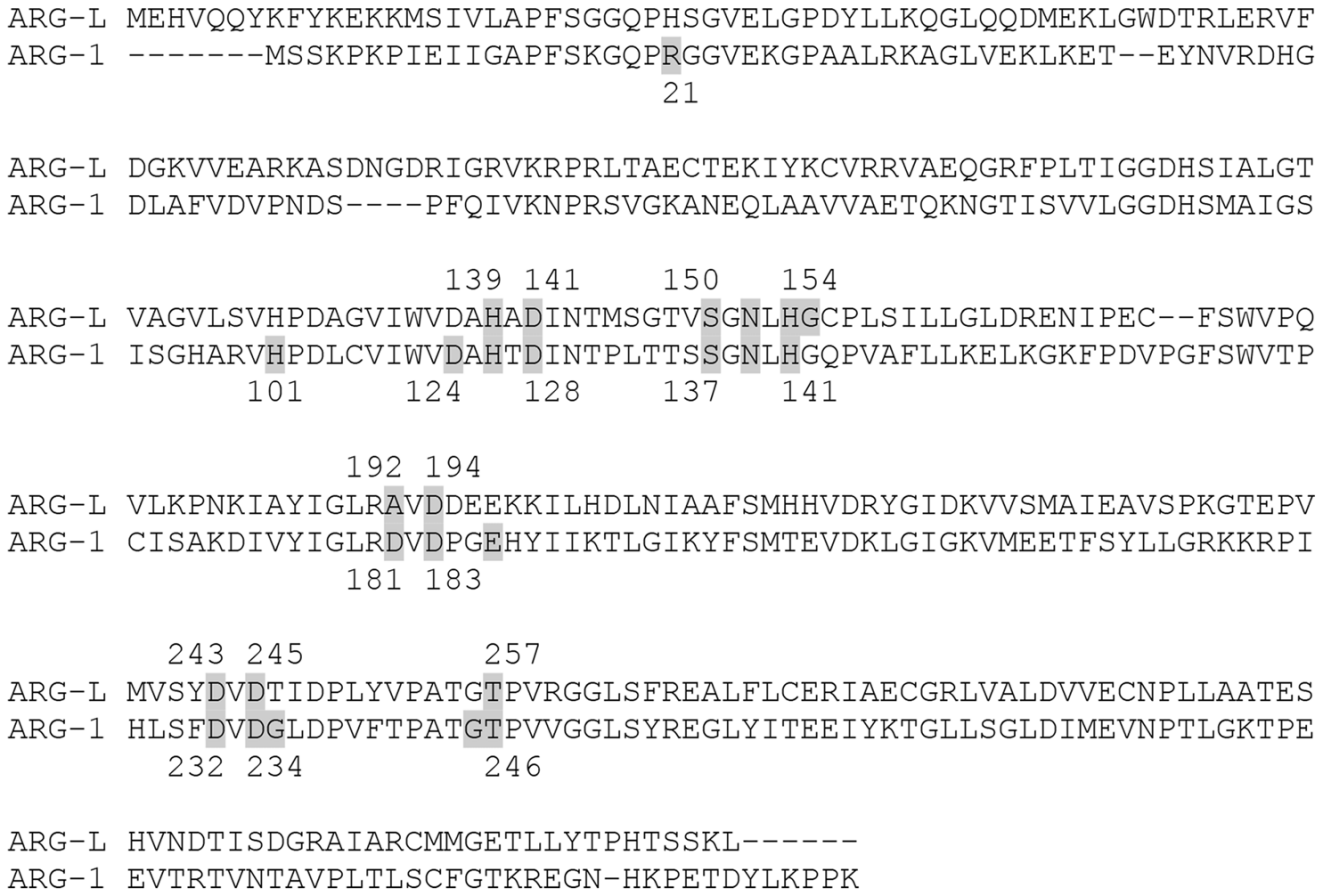
Alignment of rat liver arginase (ARG-1) with *L. (L.) amazonensis* arginase (ARG-L). Amino acids that participate in the interaction with inhibitors are marked in gray.

The compound (−)-epicatechin shows an inversion of 180° in interactions with different arginases, which can be easily visualized by observing the L-serine residue. Ser150 (ARG-L) receives the H-bond of the catechol group, whereas the equivalent Ser137 (ARG-1) donates an H-bond to the hydroxyl in the double ring of the flavanol (Figure 4). The best complex net of interaction occurs with EGCG and ARG-L and involves twelve H-bonds and hydrophobic interactions with two L-histidine residues (His154 and His139). EGCG forms nine H-bonds with ARG-1 but with different and nonequivalent amino acids compared to ARG-L (Figure 5). Gallic acid forms four H-bonds with ARG-L through the interaction with Ala192, Asp194, Asp141 and Gly155 (Figure 6). A comparison of the primary structures of ARG-L and ARG-1 show the equivalent amino acids that interact with the inhibitors (Figure 7).

The docking of the compounds has shown the differences of their interactions with the amino acid residues of ARG-L and ARG-1 that can explain the greater inhibition of ARG-L. The hydrogen bonding, the molecular weights and the number of free atom-atom bond torsions (entropic contribution) are related to the docking score energies. Consequently, those features should be considered carefully when designing of new inhibitors.

## Discussion

Enzymes and constitutive proteins from the glycosome, a unique, compartmentalized organelle in trypanosomatids, are promising drug targets [28]. It was demonstrated previously that flavonoids, including fisetin, luteolin and quercetin and derived glucoside compounds, are potent ARG-L inhibitors [20], [29].

Arginase was found to be increased in lesions of patients with cutaneous leishmaniasis [16] and in patients with visceral leishmaniasis and HIV co-infection [15]. The arginase from peripheral blood mononuclear cells was suggested as a marker of disease status in patients with visceral leishmaniasis due to *Leishmania (Leishmania) donovani* [17].

The habitual, low intake of cocoa, which contains flavanols, including (−)-epicatechin, efficiently reduced blood pressure and improved NO biosynthesis [30]. The NO products (S-nitrosothiols, nitrite and nitrate) were increased by (−)-epicatechin in healthy men [31]. These effects of flavanols ameliorated endothelial function, increasing NO and decreasing arginase activity by down regulating the transcription of the mRNA for arginase [32].

Development of drugs to be used as antileishmanial compounds has not resulted in safe and inexpensive therapies. Leishmaniasis primarily affects people who do not have access to high caloric diets or to food containing flavonols (e.g., quercetin and luteolin) and flavanols (e.g., EGCG and (−)-epicatechin) present in tropical fruits, cacao, onion and broccoli, for example.

In this study, we demonstrated that the flavanols (+)-catechin, (−)-epicatechin and EGCG are strong inhibitors of ARG-L and in high concentrations also inhibit ARG-1. In *L. (L.) amazonensis* EGCG damages mitochondria, contributing to the death of the parasite [4]. EGCG also shows leishmanicidal activity against *L. (L.) donovani* amastigotes, but (+)-catechin and (−)-epicatechin have little effect on the growth of the parasite [5]. In this study, it has been determined that (+)-catechin and (−)-epicatechin are competitive inhibitors of ARG-L, while EGCG is a mixed inhibitor and gallic acid is a noncompetitive inhibitor. The cytoplasmic vacuolization observed in *L. (L.) amazonensis* after EGCG treatment [4] was also observed after treatment of parasite promastigotes of the same species with flavonoids enriched in arginase inhibitors present in extracts of the leaves of the plant *Cecropia pachystachya* [33].

In addition, it has been shown that flavanols have weak inhibition activity against ARG-1. This study indicated that the principal activity of flavan-3-ol (−)-epicatechin and flavanol metabolites is to increase NO biosynthesis by decreasing the mRNA of the endothelial arginase [32] rather than to inhibit endothelial arginase directly. These results also indicated that mammal and parasite arginases are different and that specific inhibitors for killing parasites can be designed and developed.

Docking analyses showed that the interactions of the arginase inhibitors tested with the active sites of ARG-L and ARG-1 involved different amino acids, and the best poses obtained by flexible docking were different. The interactions observed in the ARG-L/EGCG complex can explain the mixed inhibition of ARG-L by EGCG. In contrast, understanding the binding of gallic acid with ARG-L through the interaction with Ala192, Asp194, Asp141 and Gly155 could lead to the design of potent noncompetitive inhibitors (Figure 6). The differences in position of the four compounds tested in the active sites of the arginases can explain the stronger inhibition of ARG-L compared to ARG-1 and also contribute to designing specific inhibitors of ARG-L.

In conclusion, this work has shown that selective, mixed inhibition of arginase by EGCG helps to explain its mechanism of action against *L. (L.) amazonensis*. The ARG-L inhibitors EGCG and (−)-epicatechin, which increase NO by down regulation of human arginase, can contribute to leishmaniasis treatment in association with current antileishmanial agents.
